# Draft Genome Sequence of the Antarctic Psychrophilic Bacterium *Pseudomonas syringae* Strain Lz4W

**DOI:** 10.1128/genomeA.00377-13

**Published:** 2013-06-20

**Authors:** Apuratha Pandiyan, Malay K. Ray

**Affiliations:** CSIR-Centre for Cellular and Molecular Biology, Hyderabad, India

## Abstract

The psychrophilic bacterium *Pseudomonas syringae* strain Lz4W was isolated from soil samples from Antarctica to decipher the mechanisms of low-temperature adaptation. We report here the 4.982-Mb draft genome sequence of *P. syringae* Lz4W. This sequence will provide insights into the genomic basis of the psychrophilicity of this bacterium.

## GENOME ANNOUNCEMENT

Antarctic psychrophilic bacteria grow at and around 0°C (1, 2). The molecular mechanisms by which these bacteria overcome barriers for growth at low temperatures are not well understood. To investigate these mechanisms, we have been using *Pseudomonas syringae* strain Lz4W as a model system (3, 4, 5). This Gram-negative nonfluorescent pseudomonad was isolated from soil samples from the Schirmacher Oasis and Antarctica (6). Our studies have yielded novel insights into various mechanisms of psychrophilic adaptation that include modifications in lipopolysaccharides (7, 8), the RecBCD complex (9, 10, 11), RNA polymerase (12, 13), and a novel RNA degradosome with exoribonuclease RNase R (14, 15, 16). We and others have generated a transposon-mutagenized library of cold-sensitive mutants which do not grow at 4°C but grow at 22°C, and their suppressors, for characterization of essential genes for growth at low temperatures (9, 17; M. K. Ray, unpublished results). In further explorations we have determined the genome sequence of the bacterium.

We sequenced the genomes of the wild type (WT) and a suppressor of the *recBCD* mutant (LCBD) of *P. syringae* Lz4W (11). Genomic DNAs were sequenced using the Illumina GAIIx sequencing system. Two genomic data sets produced about 63.9 and 79.6 million paired-end reads of 76 nucleotides (nt) in an ~350-bp insert library of the WT and the mutant, respectively. All the reads were assembled using Velvet v1.2.03 (18), employing a hash length (k-mer) of 57 nt. The assembly produced >80-fold genome coverage. For the WT, the assembly generated 441 contigs with an *N*_50_ length of 82,141 bp (maximum contig length, 272,987 bp). On the other hand, the genome assembly for the mutant contained 291 contigs with an *N*_50_ of 149,002 bp (maximum length, 455,988 bp). The tetracycline-cassette-disrupted *recCBD* operon and the Tn*5* transposon were identified within the sequences of the LCBD suppressor mutant. Contigs of the two genomes were aligned using CodonCode Aligner v4.0.3 (CodonCode, Deadham, MA) to fill gaps and expand the assembly. This led to the generation of 72 scaffolds representing the whole genome. Subsequently, nonspecific nucleotides (N) in the scaffolds were replaced by correct nucleotides, which were determined by PCR amplification and sequencing. Additionally, two kinds of rRNA operons were characterized by the presence and absence of two tRNA genes (tRNA^Ile^ and tRNA^Ala^) in the intergenic spacer between the 16S and 23S rRNA genes by PCR amplification and sequencing. Altogether, the final assembly of the wild-type genome produced 4,982,906 bp in 42 contigs, with an *N*_50_ contig length of 236,678 bp (maximum, 804,687 bp) and a 58.67% G+C content.

The genome sequence was annotated using RAST (19) and the NCBI Prokaryotic Genomes Automatic Annotation Pipeline (PGAAP) (20). The annotation predicted 4,450 protein-encoding genes, 62 tRNA-encoding genes, and 6 genes for three rRNAs (16S, 23S, and 5S) on two separate contigs. The sequence analysis suggests that *P. syringae* Lz4W is more closely related to *P. fluorescens* than to plant-pathogenic *P. syringae* species and therefore should be classified as a distinct new species under the genus *Pseudomonas*, which will be reported separately.

### Nucleotide sequence accession numbers.

This Whole-Genome Shotgun project has been deposited at DDBJ/EMBL/GenBank under the accession number AOGS00000000. The version described in this paper is the first version, number AOGS01000000.
